# Crystal structure of ethyl 2-{4-[(2-oxo-3-phenyl-1,2-di­hydro­quinoxalin-1-yl)meth­yl]-1*H*-1,2,3-triazol-1-yl}acetate

**DOI:** 10.1107/S2414314622006939

**Published:** 2022-07-14

**Authors:** Nadeem Abad, Mohcine Missioui, Abdulsalam Alsubari, Joel T. Mague, El Mokhtar Essassi, Youssef Ramli

**Affiliations:** a Laboratory of Medicinal Chemistry, Drug Sciences Research Center, Faculty of Medicine and Pharmacy, Mohammed V University in Rabat, Morocco; b Laboratory of Heterocyclic Organic Chemistry, Faculty of Sciences, Mohammed V University in Rabat, Morocco; c Laboratory of Medicinal Chemistry, Faculty of Clinical Pharmacy, 21 September University, Yemen; d Department of Chemistry, Tulane University, New Orleans, LA 70118, USA; University of Antofagasta, Chile

**Keywords:** crystal structure, quinoxaline, triazole, hydrogen bond, π-stacking

## Abstract

The quinoxaline portion of the title mol­ecule, C_21_H_19_N_5_O_3_, is not quite planar as indicated by a dihedral angle of 3.38 (7)° between the constituent rings. The mol­ecule is ‘U-shaped’, which is consolidated by an intra­molecular anti­parallel carbonyl electrostatic inter­action with C··O distances of 2.8905 (16) and 3.0221 (15) Å, in the crystal forms corrugated layers through C—H⋯O and C—H⋯N hydrogen bonds and C—H⋯π(ring) and π-stacking inter­actions.

## Abstract 2

The quinoxaline portion of the title mol­ecule is not quite planar and the conformation of the entire mol­ecule is ‘U-shaped’, which is consolidated by an intra­molecular anti­parallel carbonyl electrostatic inter­action. In the crystal, the mol­ecules form corrugated layers through C—H⋯O and C—H⋯N hydrogen bonds and C—H⋯π(ring) and π-stacking inter­actions.

## Graphical abstract

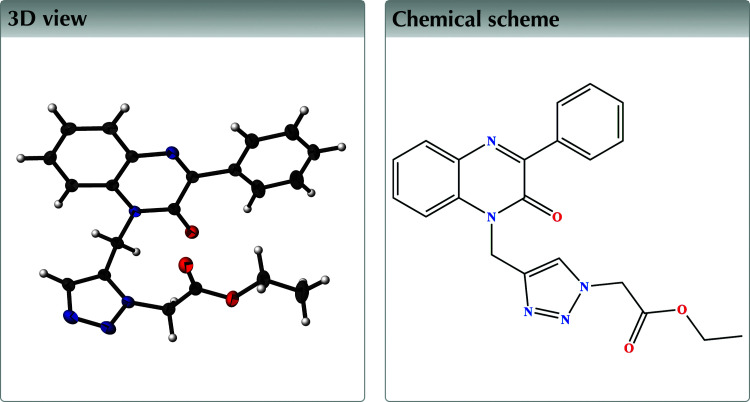

## Structure description

**Figure 1:**
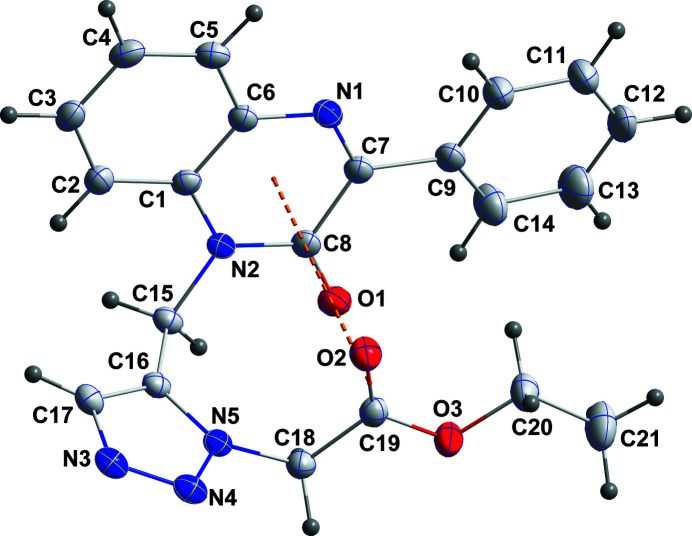
The title mol­ecule with the labeling scheme and 50% probability ellipsoids. The π inter­action between the C19=O2 carbonyl group and the C1/C6/N1/C7/C8/N2 ring is shown by an orange dashed line.

Quinoxaline derivatives exhibit a wide range of biological applications including anti­microbial (Teja *et al.*, 2016 ▸), anti-inflammatory (Guirado *et al.*, 2012 ▸), anti­cancer (Abbas *et al.*, 2015 ▸), anti­diabetic (Kulkarni *et al.*, 2012 ▸) and anti­histaminic (Sridevi *et al.*, 2010 ▸) effects. As a continuation of our research on the synthesis and biological properties of quinoxaline derivatives (Missioui *et al.*, 2022*a* ▸,*b* ▸,*c* ▸), the title compound (Fig. 1 ▸) was prepared and its crystal structure is reported here.

**Figure 2:**
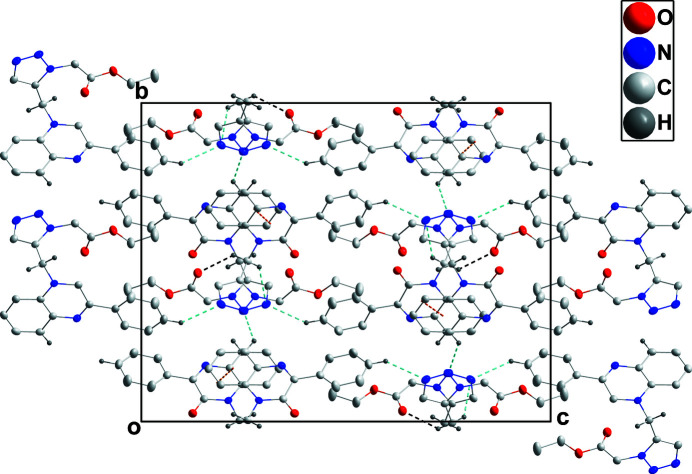
Packing viewed along the *a*-axis direction. C—H⋯O and C—H⋯N hydrogen bonds are shown, respectively, by black and light-blue dashed lines while the π-stacking inter­actions are shown by orange dashed lines.

The quinoxaline portion is not quite planar as indicated by a dihedral angle of 3.38 (7)° between the constituent rings. The dihedral angle between the C9–C14 and C1/C6/N1/C7/C8/N2 rings is 9.05 (8)° while that between the latter ring and the triazole ring is 78.47 (3)°. The mol­ecule adopts a ‘U-shaped’ conformation, which is consolidated by an intramolecular antiparallel carbonyl electrostatic interaction (Allen *et al.*, 1998 ▸) between the C8=O1 and C19=O2 groups with C19⋯O1 = 2.890 Å and C8⋯O2 = 3.022 Å. In the crystal, C12—H12⋯N3 hydrogen bonds (Table 1 ▸) lead to the formation of chains extending along the *c*-axis direction, which are linked into corrugated layers by C5—H5⋯N4 and C15—H15*B*⋯O2 hydrogen bonds and by C15—15A⋯*Cg*1 inter­actions (Table 1 ▸ and Fig. 2 ▸). These are accompanied by weak π-stacking inter­actions between C1/C6/N1/C7/C8/N2 and C1–C6 rings related by the symmetry operation *x* − 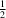 , *y*, −*z* − 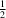 [centroid–centroid distance = 3.8105 (7) Å, dihedral angle = 6.13 (6)°].

## Synthesis and crystallization

To a solution of 3-phenyl-1-(prop-2-yn-1-yl)quinoxalin-2(1*H*)-one (0.68 mmol) in ethanol (15 ml) was added ethyl 2-azido­acetate (1.03 mmol). The reaction mixture was stirred under reflux for 72 h. After completion of the reaction (monitored by TLC), the solution was concentrated and the residue was purified by column chromatography on silica gel by using a hexa­ne/ethyl acetate mixture (9:1) as eluent. The solid product obtained was crystallized from ethanol solution to afford colorless crystals. Yield 80%, m.p. = 408–410 K. ^1^H MNR (300 MHz, CDCl_3_) δ (p.p.m.):1.22–1.26 (*t*, 3H, CH_3_, *J* = 6 Hz); 4.12-4.19 (*q*, 2H, *O*—CH_2_, *J* = 6 Hz); 5.57 (*s*, 2H, N—CH_2_CO_2_); 5.60 (s, 2*H*, *N*—CH_2_); 7.72 (*s*, H,CH_triazole_); 7.44–8.31 (*m*, 9H_arom_); ^13^C MNR (75 MHz,CDCl_3_) δ (p.p.m.):13.95 (CH_3_); 34.99 (O—CH_2_); 50.01(N—CH_2_C=O); 62.48 (N—CH_2_); 113.48, 124.61, 128.19 (traizole), 129.52, 130.70, 130.85, 131.16, 131.79, (CH_arom_); 132.79, 133.53, 134.34, 135.52, 153.69 (C_q_); 154.32 (C=O_arom_);166.80 (C=O_acetate_)

## Refinement

Crystal data, data collection and structure refinement details are summarized in Table 2 ▸.

## Supplementary Material

Crystal structure: contains datablock(s) global, I. DOI: 10.1107/S2414314622006939/bx4022sup1.cif

Structure factors: contains datablock(s) I. DOI: 10.1107/S2414314622006939/bx4022Isup2.hkl

Supporting information file. DOI: 10.1107/S2414314622006939/bx4022Isup3.cml

CCDC reference: 2184531

Additional supporting information:  crystallographic information; 3D view; checkCIF report

## Figures and Tables

**Table 1:** Hydrogen-bond geometry (Å, °) *Cg*1 is the centroid of the triazole ring.

*D*—H⋯*A*	*D*—H	H⋯*A*	*D*⋯*A*	*D*—H⋯*A*
C5—H5⋯N4^i^	0.973 (16)	2.462 (16)	3.2183 (17)	134.3 (12)
C12—H12⋯N3^ii^	0.988 (19)	2.572 (19)	3.4094 (18)	142.5 (15)
C15—H15*A*⋯*Cg*1^iii^	0.997 (16)	2.657 (15)	3.3580 (14)	127.5 (10)
C15—H15*B*⋯O2^iv^	0.986 (15)	2.464 (15)	3.2459 (16)	135.9 (11)

Symmetry codes: (i) 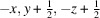 ; (ii) 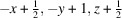 ; (iii) 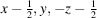 ; (iv) 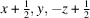 .

**Table 2:** Experimental details

Crystal data	
Chemical formula	C_21_H_19_N_5_O_3_
*M*_r_	389.41
Crystal system, space group	Orthorhombic, *P* *b* *c* *a*
Temperature (K)	150
*a*, *b*, *c* (Å)	8.8585 (3), 18.0405 (5), 23.1961 (7)
*V* (Å^3^)	3707.0 (2)
*Z*	8
Radiation type	Cu *K*α
μ (mm^−1^)	0.79
Crystal size (mm)	0.21 × 0.10 × 0.02

Data collection	
Diffractometer	Bruker D8 VENTURE PHOTON 100 CMOS
Absorption correction	Multi-scan (*SADABS*; Krause *et al.*, 2015 ▸)
*T*_min_, *T*_max_	0.89, 0.98
No. of measured, independent and observed [*I* > 2σ(*I*)] reflections	26694, 3662, 3086
*R*_int_	0.047
(sin θ/λ)_max_ (Å^−1^)	0.618

Refinement	
*R*[*F* ^2^ > 2σ(*F* ^2^)], *wR*(*F* ^2^), *S*	0.035, 0.087, 1.05
No. of reflections	3662
No. of parameters	339
H-atom treatment	All H-atom parameters refined
Δρ_max_, Δρ_min_ (e Å^−3^)	0.24, −0.21

Computer programs: *APEX3* and *SAINT* (Bruker, 2016 ▸), *SHELXT* (Sheldrick, 2015*a* ▸), *SHELXL2018/1* (Sheldrick, 2015*b* ▸), *DIAMOND* (Brandenburg & Putz, 2012 ▸) and *SHELXTL* (Sheldrick, 2008 ▸).

## full crystallographic data

### Crystal data

**Table:**

C_21_H_19_N_5_O_3_	*D*_x_ = 1.395 Mg m^−^^3^
*M**_r_* = 389.41	Cu *K*α radiation, λ = 1.54178 Å
Orthorhombic, *P**b**c**a*	Cell parameters from 9932 reflections
*a* = 8.8585 (3) Å	θ = 3.8–72.3°
*b* = 18.0405 (5) Å	µ = 0.79 mm^−^^1^
*c* = 23.1961 (7) Å	*T* = 150 K
*V* = 3707.0 (2) Å^3^	Plate, colourless
*Z* = 8	0.21 × 0.10 × 0.02 mm
*F*(000) = 1632	

### Data collection

**Table:**

Bruker D8 VENTURE PHOTON 100 CMOS diffractometer	3662 independent reflections
Radiation source: INCOATEC IµS micro–focus source	3086 reflections with *I* > 2σ(*I*)
Mirror monochromator	*R*_int_ = 0.047
Detector resolution: 10.4167 pixels mm^-1^	θ_max_ = 72.5°, θ_min_ = 3.8°
ω scans	*h* = −10→10
Absorption correction: multi-scan (*SADABS*; Krause *et al.*, 2015)	*k* = −22→22
*T*_min_ = 0.89, *T*_max_ = 0.98	*l* = −28→27
26694 measured reflections	

### Refinement

**Table:**

Refinement on *F*^2^	Secondary atom site location: difference Fourier map
Least-squares matrix: full	Hydrogen site location: difference Fourier map
*R*[*F*^2^ > 2σ(*F*^2^)] = 0.035	All H-atom parameters refined
*wR*(*F*^2^) = 0.087	*w* = 1/[σ^2^(*F*_o_^2^) + (0.0384*P*)^2^ + 1.2273*P*] where *P* = (*F*_o_^2^ + 2*F*_c_^2^)/3
*S* = 1.05	(Δ/σ)_max_ < 0.001
3662 reflections	Δρ_max_ = 0.24 e Å^−^^3^
339 parameters	Δρ_min_ = −0.21 e Å^−^^3^
0 restraints	Extinction correction: *SHELXL 2018/1* (Sheldrick, 2015*b*), Fc^*^=kFc[1+0.001xFc^2^λ^3^/sin(2θ)]^-1/4^
Primary atom site location: structure-invariant direct methods	Extinction coefficient: 0.00064 (5)

### Special details

**Table:**

Geometry. All esds (except the esd in the dihedral angle between two l.s. planes) are estimated using the full covariance matrix. The cell esds are taken into account individually in the estimation of esds in distances, angles and torsion angles; correlations between esds in cell parameters are only used when they are defined by crystal symmetry. An approximate (isotropic) treatment of cell esds is used for estimating esds involving l.s. planes.
Refinement. Refinement of F^2^ against ALL reflections. The weighted R-factor wR and goodness of fit S are based on F^2^, conventional R-factors R are based on F, with F set to zero for negative F^2^. The threshold expression of F^2^ > 2sigma(F^2^) is used only for calculating R-factors(gt) etc. and is not relevant to the choice of reflections for refinement. R-factors based on F^2^ are statistically about twice as large as those based on F, and R- factors based on ALL data will be even larger.All H atom positional and Uiso values were freely refined.

### Fractional atomic coordinates and isotropic or equivalent isotropic displacement parameters (Å^2^)

**Table:**

	*x*	*y*	*z*	*U*_iso_*/*U*_eq_	
O1	0.43111 (11)	0.52576 (5)	0.37440 (4)	0.0275 (2)	
O2	0.09359 (10)	0.46632 (5)	0.36206 (4)	0.0270 (2)	
O3	0.20937 (11)	0.40033 (5)	0.43166 (4)	0.0303 (2)	
N1	0.18289 (12)	0.67590 (6)	0.34317 (5)	0.0226 (2)	
N2	0.34767 (11)	0.56638 (5)	0.28730 (4)	0.0193 (2)	
N3	0.16535 (13)	0.36820 (6)	0.19501 (5)	0.0252 (2)	
N4	0.16515 (13)	0.34789 (6)	0.24943 (5)	0.0250 (2)	
N5	0.26117 (12)	0.39291 (6)	0.27772 (5)	0.0208 (2)	
C1	0.26318 (14)	0.61609 (6)	0.25427 (5)	0.0195 (3)	
C2	0.26183 (16)	0.61555 (7)	0.19390 (6)	0.0238 (3)	
H2	0.3228 (19)	0.5802 (9)	0.1724 (7)	0.034 (4)*	
C3	0.17264 (16)	0.66602 (7)	0.16465 (6)	0.0264 (3)	
H3	0.1743 (18)	0.6650 (9)	0.1222 (7)	0.033 (4)*	
C4	0.08259 (16)	0.71695 (7)	0.19408 (6)	0.0260 (3)	
H4	0.0164 (19)	0.7523 (9)	0.1731 (7)	0.034 (4)*	
C5	0.08467 (15)	0.71842 (7)	0.25326 (6)	0.0236 (3)	
H5	0.0237 (18)	0.7530 (9)	0.2755 (7)	0.031 (4)*	
C6	0.17717 (14)	0.66902 (7)	0.28405 (5)	0.0207 (3)	
C7	0.26837 (14)	0.63234 (7)	0.37345 (5)	0.0211 (3)	
C8	0.35681 (14)	0.57104 (7)	0.34670 (5)	0.0208 (3)	
C9	0.27185 (16)	0.64586 (7)	0.43697 (6)	0.0248 (3)	
C10	0.1715 (2)	0.69806 (8)	0.45951 (6)	0.0356 (3)	
H10	0.101 (2)	0.7214 (11)	0.4333 (8)	0.053 (6)*	
C11	0.1707 (2)	0.71453 (9)	0.51787 (7)	0.0435 (4)	
H11	0.098 (2)	0.7522 (12)	0.5330 (9)	0.062 (6)*	
C12	0.2704 (2)	0.67988 (9)	0.55500 (6)	0.0408 (4)	
H12	0.268 (2)	0.6896 (11)	0.5969 (8)	0.050 (5)*	
C13	0.3689 (2)	0.62824 (10)	0.53366 (7)	0.0434 (4)	
H13	0.442 (2)	0.6034 (12)	0.5596 (9)	0.063 (6)*	
C14	0.37072 (19)	0.61077 (9)	0.47502 (7)	0.0369 (4)	
H14	0.443 (2)	0.5717 (11)	0.4606 (8)	0.051 (5)*	
C15	0.42864 (14)	0.50430 (7)	0.25935 (6)	0.0206 (3)	
H15A	0.5028 (17)	0.4856 (8)	0.2882 (6)	0.025 (4)*	
H15B	0.4831 (17)	0.5225 (8)	0.2251 (6)	0.022 (4)*	
C16	0.32363 (14)	0.44361 (6)	0.24135 (5)	0.0191 (3)	
H16	0.2784 (19)	0.4473 (9)	0.1516 (7)	0.036 (4)*	
C17	0.26194 (15)	0.42622 (7)	0.18895 (6)	0.0226 (3)	
C18	0.28965 (15)	0.37793 (7)	0.33815 (6)	0.0232 (3)	
H18A	0.3932 (19)	0.3894 (9)	0.3470 (7)	0.030 (4)*	
H18B	0.2764 (17)	0.3237 (9)	0.3441 (6)	0.026 (4)*	
C19	0.18500 (14)	0.42063 (7)	0.37736 (5)	0.0221 (3)	
C20	0.11923 (18)	0.43913 (9)	0.47469 (6)	0.0329 (3)	
H20A	0.1346 (19)	0.4932 (10)	0.4678 (7)	0.036 (4)*	
H20B	0.0111 (19)	0.4247 (9)	0.4681 (7)	0.032 (4)*	
C21	0.1730 (2)	0.41458 (12)	0.53292 (7)	0.0456 (4)	
H21A	0.165 (2)	0.3587 (12)	0.5382 (9)	0.062 (6)*	
H21B	0.111 (2)	0.4381 (11)	0.5639 (9)	0.057 (6)*	
H21C	0.277 (2)	0.4295 (11)	0.5392 (8)	0.052 (6)*	

### Atomic displacement parameters (Å^2^)

**Table:**

	*U*^11^	*U*^22^	*U*^33^	*U*^12^	*U*^13^	*U*^23^
O1	0.0328 (5)	0.0238 (5)	0.0258 (5)	0.0042 (4)	−0.0065 (4)	0.0000 (4)
O2	0.0241 (5)	0.0317 (5)	0.0251 (5)	0.0058 (4)	−0.0013 (4)	0.0000 (4)
O3	0.0350 (5)	0.0339 (5)	0.0219 (5)	0.0116 (4)	0.0037 (4)	0.0036 (4)
N1	0.0247 (5)	0.0210 (5)	0.0222 (5)	0.0002 (4)	−0.0005 (5)	−0.0029 (4)
N2	0.0204 (5)	0.0169 (5)	0.0204 (5)	−0.0001 (4)	−0.0007 (4)	−0.0021 (4)
N3	0.0228 (6)	0.0248 (6)	0.0280 (6)	0.0015 (4)	−0.0014 (5)	−0.0069 (4)
N4	0.0232 (5)	0.0218 (5)	0.0301 (6)	−0.0013 (4)	−0.0003 (5)	−0.0062 (4)
N5	0.0212 (5)	0.0189 (5)	0.0224 (5)	−0.0007 (4)	0.0010 (4)	−0.0026 (4)
C1	0.0197 (6)	0.0167 (5)	0.0222 (6)	−0.0030 (5)	−0.0013 (5)	0.0005 (5)
C2	0.0270 (7)	0.0221 (6)	0.0223 (6)	−0.0021 (5)	0.0022 (5)	−0.0002 (5)
C3	0.0328 (7)	0.0249 (6)	0.0215 (7)	−0.0052 (6)	−0.0021 (6)	0.0031 (5)
C4	0.0280 (7)	0.0198 (6)	0.0302 (7)	−0.0029 (5)	−0.0068 (6)	0.0041 (5)
C5	0.0230 (6)	0.0184 (6)	0.0295 (7)	−0.0002 (5)	−0.0029 (5)	−0.0013 (5)
C6	0.0219 (6)	0.0185 (6)	0.0216 (6)	−0.0028 (5)	−0.0010 (5)	−0.0014 (5)
C7	0.0231 (6)	0.0189 (6)	0.0213 (6)	−0.0031 (5)	−0.0003 (5)	−0.0013 (5)
C8	0.0217 (6)	0.0195 (6)	0.0213 (6)	−0.0032 (5)	−0.0016 (5)	−0.0012 (5)
C9	0.0306 (7)	0.0215 (6)	0.0225 (7)	−0.0046 (5)	−0.0001 (6)	−0.0022 (5)
C10	0.0537 (10)	0.0285 (7)	0.0246 (7)	0.0063 (7)	0.0022 (7)	−0.0013 (6)
C11	0.0717 (12)	0.0329 (8)	0.0260 (8)	0.0098 (8)	0.0073 (8)	−0.0043 (6)
C12	0.0684 (12)	0.0340 (8)	0.0201 (7)	−0.0059 (8)	0.0021 (7)	−0.0038 (6)
C13	0.0540 (10)	0.0499 (10)	0.0263 (8)	0.0030 (8)	−0.0102 (7)	−0.0036 (7)
C14	0.0395 (8)	0.0450 (9)	0.0263 (8)	0.0067 (7)	−0.0072 (6)	−0.0068 (6)
C15	0.0179 (6)	0.0198 (6)	0.0241 (6)	0.0009 (5)	0.0017 (5)	−0.0031 (5)
C16	0.0173 (5)	0.0178 (5)	0.0222 (6)	0.0026 (5)	0.0025 (5)	−0.0024 (5)
C17	0.0211 (6)	0.0240 (6)	0.0226 (6)	0.0029 (5)	0.0008 (5)	−0.0044 (5)
C18	0.0251 (7)	0.0219 (6)	0.0226 (7)	0.0024 (5)	0.0014 (5)	0.0008 (5)
C19	0.0219 (6)	0.0232 (6)	0.0212 (6)	−0.0020 (5)	0.0006 (5)	0.0004 (5)
C20	0.0366 (8)	0.0404 (8)	0.0218 (7)	0.0107 (7)	0.0051 (6)	0.0006 (6)
C21	0.0474 (10)	0.0652 (12)	0.0241 (8)	0.0154 (9)	0.0001 (7)	0.0041 (7)

### Geometric parameters (Å, º)

**Table:**

O1—C8	1.2301 (16)	C9—C14	1.395 (2)
O2—C19	1.2088 (16)	C9—C10	1.397 (2)
O3—C19	1.3293 (16)	C10—C11	1.386 (2)
O3—C20	1.4574 (17)	C10—H10	0.97 (2)
N1—C7	1.2978 (17)	C11—C12	1.383 (2)
N1—C6	1.3780 (16)	C11—H11	1.00 (2)
N2—C8	1.3827 (16)	C12—C13	1.369 (2)
N2—C1	1.3970 (16)	C12—H12	0.988 (19)
N2—C15	1.4797 (15)	C13—C14	1.396 (2)
N3—N4	1.3144 (16)	C13—H13	0.99 (2)
N3—C17	1.3592 (18)	C14—H14	1.01 (2)
N4—N5	1.3468 (15)	C15—C16	1.4962 (17)
N5—C16	1.3618 (16)	C15—H15A	0.997 (16)
N5—C18	1.4497 (17)	C15—H15B	0.986 (15)
C1—C2	1.4005 (18)	C16—C17	1.3691 (18)
C1—C6	1.4034 (17)	C17—H16	0.957 (17)
C2—C3	1.3832 (19)	C18—C19	1.5100 (18)
C2—H2	0.974 (17)	C18—H18A	0.963 (17)
C3—C4	1.395 (2)	C18—H18B	0.995 (16)
C3—H3	0.984 (16)	C20—C21	1.499 (2)
C4—C5	1.3732 (19)	C20—H20A	0.997 (18)
C4—H4	0.994 (17)	C20—H20B	1.005 (17)
C5—C6	1.4055 (18)	C21—H21A	1.02 (2)
C5—H5	0.973 (16)	C21—H21B	1.00 (2)
C7—C8	1.4905 (17)	C21—H21C	0.97 (2)
C7—C9	1.4937 (17)		
			
C19—O3—C20	115.32 (11)	C13—C12—C11	119.30 (14)
C7—N1—C6	120.37 (11)	C13—C12—H12	119.4 (11)
C8—N2—C1	122.62 (10)	C11—C12—H12	121.2 (11)
C8—N2—C15	117.02 (10)	C12—C13—C14	120.87 (16)
C1—N2—C15	120.34 (10)	C12—C13—H13	120.3 (12)
N4—N3—C17	108.35 (11)	C14—C13—H13	118.8 (12)
N3—N4—N5	107.40 (10)	C9—C14—C13	120.44 (15)
N4—N5—C16	111.08 (10)	C9—C14—H14	120.3 (11)
N4—N5—C18	117.95 (10)	C13—C14—H14	119.2 (11)
C16—N5—C18	130.82 (11)	N2—C15—C16	112.02 (10)
N2—C1—C2	123.27 (11)	N2—C15—H15A	106.4 (9)
N2—C1—C6	117.23 (11)	C16—C15—H15A	110.4 (9)
C2—C1—C6	119.49 (12)	N2—C15—H15B	109.8 (8)
C3—C2—C1	119.38 (12)	C16—C15—H15B	108.9 (8)
C3—C2—H2	119.8 (10)	H15A—C15—H15B	109.3 (12)
C1—C2—H2	120.8 (10)	N5—C16—C17	103.54 (11)
C2—C3—C4	121.35 (12)	N5—C16—C15	124.84 (11)
C2—C3—H3	118.0 (10)	C17—C16—C15	131.57 (12)
C4—C3—H3	120.7 (10)	N3—C17—C16	109.62 (12)
C5—C4—C3	119.61 (12)	N3—C17—H16	119.7 (10)
C5—C4—H4	119.1 (10)	C16—C17—H16	130.7 (10)
C3—C4—H4	121.3 (10)	N5—C18—C19	112.36 (11)
C4—C5—C6	120.24 (12)	N5—C18—H18A	109.3 (10)
C4—C5—H5	122.3 (9)	C19—C18—H18A	110.3 (10)
C6—C5—H5	117.4 (9)	N5—C18—H18B	107.3 (9)
N1—C6—C1	122.08 (11)	C19—C18—H18B	110.2 (9)
N1—C6—C5	118.04 (11)	H18A—C18—H18B	107.1 (13)
C1—C6—C5	119.86 (12)	O2—C19—O3	125.10 (12)
N1—C7—C8	122.05 (11)	O2—C19—C18	125.61 (12)
N1—C7—C9	116.55 (11)	O3—C19—C18	109.29 (11)
C8—C7—C9	121.40 (11)	O3—C20—C21	107.51 (13)
O1—C8—N2	120.76 (12)	O3—C20—H20A	106.6 (10)
O1—C8—C7	123.81 (12)	C21—C20—H20A	112.9 (10)
N2—C8—C7	115.41 (11)	O3—C20—H20B	107.1 (9)
C14—C9—C10	117.95 (13)	C21—C20—H20B	111.4 (9)
C14—C9—C7	124.25 (13)	H20A—C20—H20B	111.0 (14)
C10—C9—C7	117.79 (12)	C20—C21—H21A	112.3 (12)
C11—C10—C9	120.89 (15)	C20—C21—H21B	110.4 (12)
C11—C10—H10	121.1 (12)	H21A—C21—H21B	107.3 (16)
C9—C10—H10	118.0 (12)	C20—C21—H21C	110.8 (12)
C12—C11—C10	120.55 (16)	H21A—C21—H21C	108.7 (17)
C12—C11—H11	120.0 (12)	H21B—C21—H21C	107.2 (16)
C10—C11—H11	119.4 (12)		
			
C17—N3—N4—N5	0.03 (14)	N1—C7—C9—C14	−171.75 (14)
N3—N4—N5—C16	0.56 (13)	C8—C7—C9—C14	9.3 (2)
N3—N4—N5—C18	−175.36 (10)	N1—C7—C9—C10	6.99 (18)
C8—N2—C1—C2	175.04 (12)	C8—C7—C9—C10	−172.00 (13)
C15—N2—C1—C2	−6.76 (18)	C14—C9—C10—C11	0.2 (2)
C8—N2—C1—C6	−4.48 (17)	C7—C9—C10—C11	−178.63 (15)
C15—N2—C1—C6	173.72 (11)	C9—C10—C11—C12	0.5 (3)
N2—C1—C2—C3	178.84 (12)	C10—C11—C12—C13	−0.9 (3)
C6—C1—C2—C3	−1.65 (19)	C11—C12—C13—C14	0.6 (3)
C1—C2—C3—C4	−0.7 (2)	C10—C9—C14—C13	−0.5 (2)
C2—C3—C4—C5	1.5 (2)	C7—C9—C14—C13	178.27 (15)
C3—C4—C5—C6	0.1 (2)	C12—C13—C14—C9	0.1 (3)
C7—N1—C6—C1	−0.69 (19)	C8—N2—C15—C16	103.20 (13)
C7—N1—C6—C5	−178.96 (12)	C1—N2—C15—C16	−75.10 (14)
N2—C1—C6—N1	4.56 (18)	N4—N5—C16—C17	−0.90 (13)
C2—C1—C6—N1	−174.98 (12)	C18—N5—C16—C17	174.34 (12)
N2—C1—C6—C5	−177.20 (11)	N4—N5—C16—C15	176.59 (11)
C2—C1—C6—C5	3.26 (18)	C18—N5—C16—C15	−8.2 (2)
C4—C5—C6—N1	175.80 (12)	N2—C15—C16—N5	−76.76 (15)
C4—C5—C6—C1	−2.51 (19)	N2—C15—C16—C17	99.98 (15)
C6—N1—C7—C8	−3.34 (18)	N4—N3—C17—C16	−0.61 (14)
C6—N1—C7—C9	177.67 (11)	N5—C16—C17—N3	0.91 (13)
C1—N2—C8—O1	179.23 (11)	C15—C16—C17—N3	−176.34 (12)
C15—N2—C8—O1	0.97 (17)	N4—N5—C18—C19	−92.47 (13)
C1—N2—C8—C7	0.80 (17)	C16—N5—C18—C19	92.57 (15)
C15—N2—C8—C7	−177.45 (10)	C20—O3—C19—O2	−1.8 (2)
N1—C7—C8—O1	−175.08 (12)	C20—O3—C19—C18	178.01 (12)
C9—C7—C8—O1	3.85 (19)	N5—C18—C19—O2	−4.81 (19)
N1—C7—C8—N2	3.29 (18)	N5—C18—C19—O3	175.42 (10)
C9—C7—C8—N2	−177.78 (11)	C19—O3—C20—C21	−174.39 (14)

### Hydrogen-bond geometry (Å, º)

*Cg*1 is the centroid of the triazole ring.

**Table:**

*D*—H···*A*	*D*—H	H···*A*	*D*···*A*	*D*—H···*A*
C5—H5···N4^i^	0.973 (16)	2.462 (16)	3.2183 (17)	134.3 (12)
C12—H12···N3^ii^	0.988 (19)	2.572 (19)	3.4094 (18)	142.5 (15)
C15—H15*A*···*Cg*1^iii^	0.997 (16)	2.657 (15)	3.3580 (14)	127.5 (10)
C15—H15*B*···O2^iv^	0.986 (15)	2.464 (15)	3.2459 (16)	135.9 (11)

Symmetry codes: (i) −*x*, *y*+1/2, −*z*+1/2; (ii) −*x*+1/2, −*y*+1, *z*+1/2; (iii) *x*−1/2, *y*, −*z*−1/2; (iv) *x*+1/2, *y*, −*z*+1/2.
